# Dystrophic changes of nigrostriatal axons harboring a Synj1 Parkinson mutation suggest catastrophic failure of endocytic mechanisms

**DOI:** 10.64898/2026.06.24.733515

**Published:** 2026-06-29

**Authors:** Yumei Wu, Peng Xu, James Moran, C. Shan Xu, Kenneth J. Hayworth, Mian Cao, Lin Shao, D. James Surmeier, Harald F. Hess, Pietro De Camilli

**Affiliations:** 1 Departments of Neuroscience and of Cell Biology Program in Cellular Neuroscience, Neurodegeneration, and Repair, Howard Hughes Medical Institute, Yale School of Medicine, New Haven, CT.; 2 Department of Neuroscience, Feinberg School of Medicine, Northwestern University, Chicago, IL 60611.; 3 Janelia Research Campus, HHMI, Ashburn, VA 20147.; 4 Center for Neurodevelopment and Plasticity, Wu Tsai Institute, Yale University, New Haven, CT,USA.; 5 Aligning Science Across Parkinson's (ASAP) Collaborative Research Network.

## Abstract

Synaptojanin 1 is a brain enriched phosphoinositide phosphatase implicated in endocytosis at the synapse. A mutation (R258Q) that selectively impairs its Sac1 phosphatase domain causes early onset familial Parkinsonism. Neurons of mice with this mutation display synaptic vesicle traffic defects across the brain, but selective dystrophic changes in a subset of dopaminergic axons in the dorsolateral striatum. Using correlative light microscopy-FIB-SEM of mutant mouse striata to visualize in 3D these abnormal structures we show that they represent clusters of focal axonal dilations harboring massive, onion-like DAT enriched plasma membrane infoldings, generally localized next to cell bodies of neighboring cells, often engulfing evaginations of such cells. This dysmorphia was associated with a deficit in dopamine release in the same striatal region. Given the involvement of Synj1 in endocytic mechanisms, these structures may reflect an imbalance between exocytosis and endocytosis. Their occurrence only in a subset of axons suggest a vulnerability threshold of these axons beyond which the expansion of the plasma membrane is not counteracted by compensatory mechanisms.

Human genetic studies have identified more than 20 genes whose mutations are responsible for Parkinson’s disease (Blauwendraat et al. 2020; Boura et al. 2025; Cogan et al. 2025). A key priority is to build on this information to elucidate disease mechanisms. One of these genes, *PARK20*, encodes Synaptojanin 1 (Synj1), a PI(4,5)P_2_ phosphatase enriched in presynaptic nerve terminals where it achieves its function through two distinct, tandemly arranged phosphatase domains - a Sac1 domain (primarily a 4-phosphatase domain) and a 5-phosphatase domain - which cleave sequentially the phosphates at the 5 and 4 position of the inositol ring of this phospholipid (McPherson et al. 1996; Cremona et al. 1999; De Camilli 2026). One of the major and best known role of Synj1 at synapses is to mediate the shedding of clathrin coats and other endocytic factors during synaptic vesicle endocytosis (Cremona et al. 1999; Verstreken et al. 2003; Wenk and De Camilli 2004; Milosevic et al. 2011), although additional roles have been reported (Gong and De Camilli 2008; Choudhry et al. 2021; Yang et al. 2022; De Camilli 2026). Mutations resulting in absence or nearly complete absence of Synj1 result in early postnatal lethality in mouse (Cremona et al. 1999) and severe childhood disorders in humans (Dyment et al. 2015; Hardies et al. 2016).

In 2013, two independent studies reported that a homozygous mutation that selectively impairs the catalytic activity of its Sac1 domain (R258Q) causes recessive familial early onset Parkinsonism (Krebs et al. 2013; Quadri et al. 2013). Since then, other *SYNJ1* mutations that only partially impairs SYNJ1’s function were found to be associated with PD (Lesage et al. 2021; Senkevich et al. 2024) but the R258Q mutation remains the best characterized. We have shown that neurons of mice homozygous for the corresponding amino acid substitution (R259Q), referred to henceforth as Synj1^RQ^ mice, display neurological manifestations reminiscent to those of human patients. This phenotype is accompanied by endocytic defects at synapses which are not restricted to dopaminergic (DAergic) neurons consistent with the housekeeping role of Synj1 (Cao et al. 2017; Cao et al. 2020; Yang et al. 2022; Ng et al. 2023). These changes were more prominent at inhibitory synapses, which typically have a high rate of spontaneous activity and thus of synaptic vesicle recycling (Cao et al. 2017). In addition, dystrophic changes were observed selectively in the dorsal striatum of these mice. They were represented by an accumulation of large abnormal foci of immunoreactivity for markers of DAergic axons, such as the plasma membrane DA transporter (DAT) and tyrosine hydroxylase (TH), which stood out among the normal fine dense network of these axons (Cao et al. 2017; Cao et al. 2020; Ng et al. 2023). Presence of these foci roughly correlated in abundance with the occurrence in the same brain regions of whorls of multilamellar structures detectable by electron microscopy (EM) (Cao et al. 2017; Ng et al. 2023). However, a correspondence between these two structures has not been proven until now.

Goal of this study was to fill this gap and to reveal the precise ultrastructure of these dystrophic axonal changes towards an understanding of disease mechanisms. To this aim we generated a Synj1^RQ^ mouse model in which DA neurons express tdTomato, thus making possible correlative light-electron microscopy (CLEM) analysis. We prove that dystrophic changes observed by light microscopy in the dorsal striatum correspond to clusters of onion-like invaginations of the axonal plasma membrane of DA axons which often engulf portions of adjacent cells. In addition, DA release in the same striatal region, particularly with repetitive stimulation, was significantly impaired. In view of the importance of Synj1 in the endocytic pathway, they may result from a chronic imbalance between addition of membrane to the plasma membrane by exocytosis and removal by endocytosis. As these changes occur only in a subset of axons and axon segments, they indicate that DA axons with impaired Synj1 function may be in a vulnerable state which in a subset of them can progress to an overt pathological state.

## RESULTS

### Generation of Synj1^RQ^ mice expressing tdTomato in DAergic neurons

**Fig. 1.**
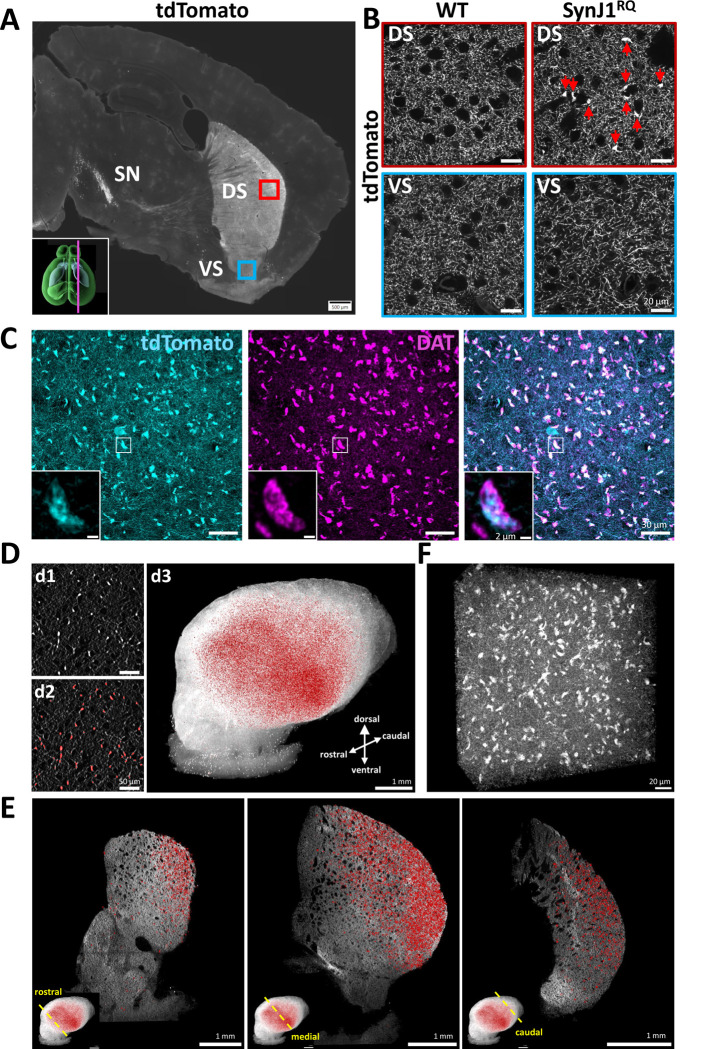
Expression of tdTomato in DAergic neurons reveal presence of dystrophic changes in a subset of their nerve terminals in the dorsal striatum of Synj1^RQ^ mice. **A**. Fluorescence image of a sagittal section of a Synj1^RQ^ mouse (4 month-old, male) expressing tdTomato under the control of a DAT promoter. tdTomato fluorescence (white) labels the entire DAergic neuronal population. SN (Substantia Nigra), DS and VS (Dorsal and Ventral Striatum). The ventral tegmental area (VTA) is not in the plane of the section. **B**. High power view of the tdTomato fluorescence in the DS and VS (regions outlined by small squares in field A). In all four fields a punctate pattern of tdTomato fluorescence, reflecting the fine arborization of DAergic axons is visible. In addition, large clusters of fluorescence are visible selectively in the dorsolateral striatum of the Synj1^RQ^ mouse brain. **C**. High power view of a region in the DLS of a Synj1^RQ^ striatum (3 month-old, male) showing colocalization of tdTomato fluorescence and DAT immunoreactivity. The inset shows that, when viewed at high magnification, the tdTomato fluorescence (a cytosolic protein) and immunoreactivity for the membrane protein DAT do not precisely overlap as expected. **D.** light sheet imaging of tdTomato fluorescence in the striatum of a delipidated td-Synj1^RQ^ whole mouse brain (3 month-old, male). **d1** and **d2**. Representative image of a region of the dorsal striatum (d1) and segmentation (red) of the bright tdTomato puncta visible in d1. **d3**. Low maginification side view of the delipidated striatum (visualized by the tdTomato fluorescence, white) showing that segmented dystrophic DAergic axons (red spots) are highly enriched in the DLS. The 3D view is shown in movie S1. **E**. Coronal sections at three different rostro-caudal positions of the striatum in **D** (d3) showing the spatial heterogeneity in the distribution of red tdTomato puncta representing DAergic dystrophic axons (see movie S2). **F.** Confocal microscopy image showing the very high abundance of large bright tdTomato foci, representing dystrophic axons, in a 217×217×100 μm^3^ volume from a region of the DLS most enriched in such foci (see movie S3). Scale bars: **A**, 500 μm; **B**, 20 μm; **C**, 30 μm, inset, 2 μm; D, d1–2, 50 μm, d3, 1 mm; **E**, 1mm; **F**, 20 μm.

To generate mice where the entire DAergic axonal network in the striatum could be selectively visualized by fluorescence microscopy without manipulations that would perturb ultrastructure (such as detergent-based membrane permeabilization required for immunofluorescence), we interbred previously described knock-in mice harboring the Synj1^RQ^ mutation (Cao et al. 2017), or WT mice as controls, with Ai9 mice which express tdTomato after Cre-dependent recombination (Madisen et al. 2010) and with DAT^IREScre^ mice which express Cre under the control of the plasma membrane dopamine transporter DAT (Bäckman et al. 2006). The resulting control and mutant mice (DAT^IRES^cre; Ai9; WT or *Synj1*^RQ/RQ^ mice) (age ranging from 1.5 to 15 months) were anesthetized and transcardially perfused by an aldehyde-based fixative followed by generation of brain vibrotome sections and whole brain clarity. As expected, inspection of these sections by fluorescence microscopy revealed a bright labeling of DA neurons (Fig. 1A). tdTomato fluorescence decorated all compartments of these neurons, including cell bodies with their dendrites in the Substantia Nigra and Ventral Tegmental area (VTA), as well as their axons which terminate with a massive highly branched arbor in the Dorsal Striatum, Nucleus Accumbens and Olfactory Tubercle.

### Abnormal foci of tdTomato fluorescence selectively in the dorsolateral striatum (DLS) of Synj1^RQ^ mutant mice

At high power view, tdTomato fluorescence in the striatum was accounted for by a fine reticulum of axonal arborization (Fig. 1B). In addition, however, sparse larger tdTomato positive foci stood out among the thinner fine axonal reticular network in the dorsal striatum of Synj1^RQ^ mice but not of WT mice. As shown by anti-DAT immunofluorescence, these foci were positive for DAT immunoreactivity (Fig. 1C), confirming that they represent dilation of DAergic axons, in agreement with previous results (Ng et al. 2023; Lin et al. 2026). Thus, these mice represent a good model system to study dystrophic changes induced by the Synj1^RQ^ mutation.

To obtain a global view of the localization of these abnormal Dopaminergic axon segments within the striatum, we delipidated the entire brain (Park et al. 2018) and then analyzed it by light sheet microscopy. Large foci of fluorescence were automatically segmented, pseudocolored in red and then superimposed on the total tdTomato fluorescence of the striatum shown in white (Fig. 1D). 3D views of the entire striatum, which are shown in Fig. 1D (field d3, a side view) and in movie S1, demonstrate the striking and selective accumulation of the bright foci in its dorsolateral region (DLS) and their near absence in the ventral striatum. This localization, with a decreasing density of foci in the lateral to medial direction, is further illustrated by virtual optical coronal planes at three different rostro-caudal positions of the striatum in Fig. 1E and movie S2. The high concentration of fluorescent foci in the most lateral region of the DLS is shown in a high power view of a small region of a 100 μm-thick vibratome section obtained from this region (Fig. 1F and movie S3).

### The Synj1^RQ^ mutation was associated with a deficit in DA release, specifically within the DLS

**Fig. 2.**
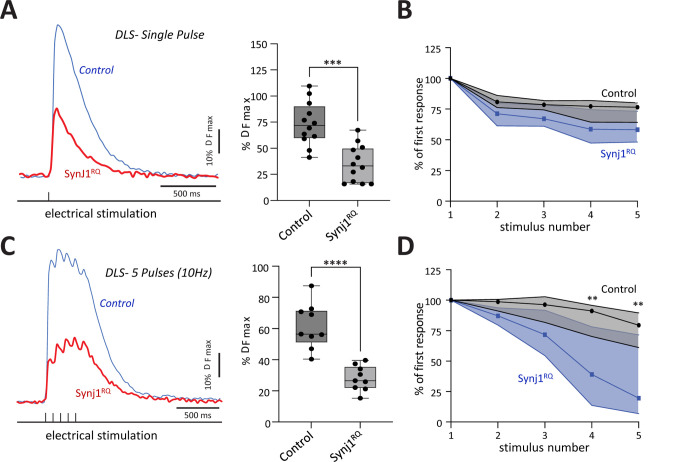
Reduced evoked DA release capacity in the dorsolateral striatum (DLS) of Synj1^RQ^ mice. **A.** Representative traces of DA release as assessed by dLigth1.3b in the DLS evoked from single-pulse stimulation. Traces are normalized to dF max (left). Quantification of the amount of DA released from terminals innervating the DLS in response to single stimulatory events. Quantifications are presented as a percentage of dF max (achieved through bath application of 100 μM DA). Synj1^RQ^: n=12, N=6; *Synj1*^+/RQ^ (control): n=12, N=7. (right). **B.** Plot illustrating mild deficits in response to consecutive single pulses in the DLS of homozygous Synj1^RQ^ mice when release is evoked by a single pulse. Responses are normalized to the first response in the stimulation protocol. Synj1^RQ^: n=11, N=4; *Synj1*^+/RQ^ (control): n=14, N=4.. **C.** Representative trace of DA release in the DLS in response to 5-pulse stimulation at 10 Hz. Traces are normalized to dF max (left). Quantification of the amount of DA released from terminals innervating the DLS in response to a 5-pulse train at 10 Hz. Quantifications are based on the dF of the 5^th^ pulse in the train and are presented as a percentage of dF max. Synj1^RQ^: n=9, N=3; *Synj1*^+/RQ^ (control): n=9, N=4. (right). **D.** Plot illustrating profound deficits in vesicle recycling in the DLS of SJ1^RQ^ mice when release is evoked a 5-pulse train at 10 Hz. Responses are normalized to the first response in the stimulation protocol. Synj1^RQ^: n=11, N=3; *Synj1*^+/RQ^ (control): n=11, N=4.

To determine whether the occurrence of structural changes of DAergic axons in the DLS was accompanied by functional changes, we used the genetically encoded sensor dLight1.3b to explore potential defects of the mutation on DA secretion. dLight1.3b was expressed using a viral vector in the DLS of Synj1^RQ^ mice and two photon laser scanning microscopy (2PLSM) was used to estimate electrically evoked DA release. In *ex vivo* brain slices from Synj1^RQ^ mice, there was a pronounced reduction in DA release in response to a single stimulus compared to DA release detected in slices of heterozygous littermates controls (*Synj1*^+/RQ^) (39% reduction, *P* = 0.0001) (Fig. 2A). Comparable reductions in release were observed following a train of five stimuli at 10 Hz (30% reduction, *P* < 0.0001) (Fig. 2C). dLight1.3b was also expressed in the ventral striatum. In contrast to the results observed in the DLS, DA release in the ventral striatum was not significantly altered (Fig. S1A–1D).

The electrical stimulation (either a single pulse or a five-pulse burst) was also repeated at ten second intervals and the changes in DA release monitored. In slices from Synj1^RQ^ mice, there was a pronounced, progressive drop in DA release evoked in response to the burst stimulation, in contrast to the response in slices from heterozygous controls (*Synj1*^+/RQ^) (Fig. 2D). The release evoked by a single stimulus trended in the same direction but did not reach statistical significance relative to *Synj1*^+/RQ^ controls (Fig. 2B). No difference was observed in the Nucleus Accumbens (NAc) of the ventral striatum in response to the same stimulation patterns (Fig. S2B and D). Together, these data suggest that SynJ1 dysfunction impairs both the initial release of DA and the response to sequential stimuli in the DLS, but not in the ventral striatum, paralleling the localization of dystrophic axon terminals only in the DLS. These results, together with the morphological observations, indicate that the effects of the Synj1 mutation preferentially impact DAergic terminals in the DLS.

### Dystrophic axonal structures are generally adjacent to cell bodies.

**Fig 3.**
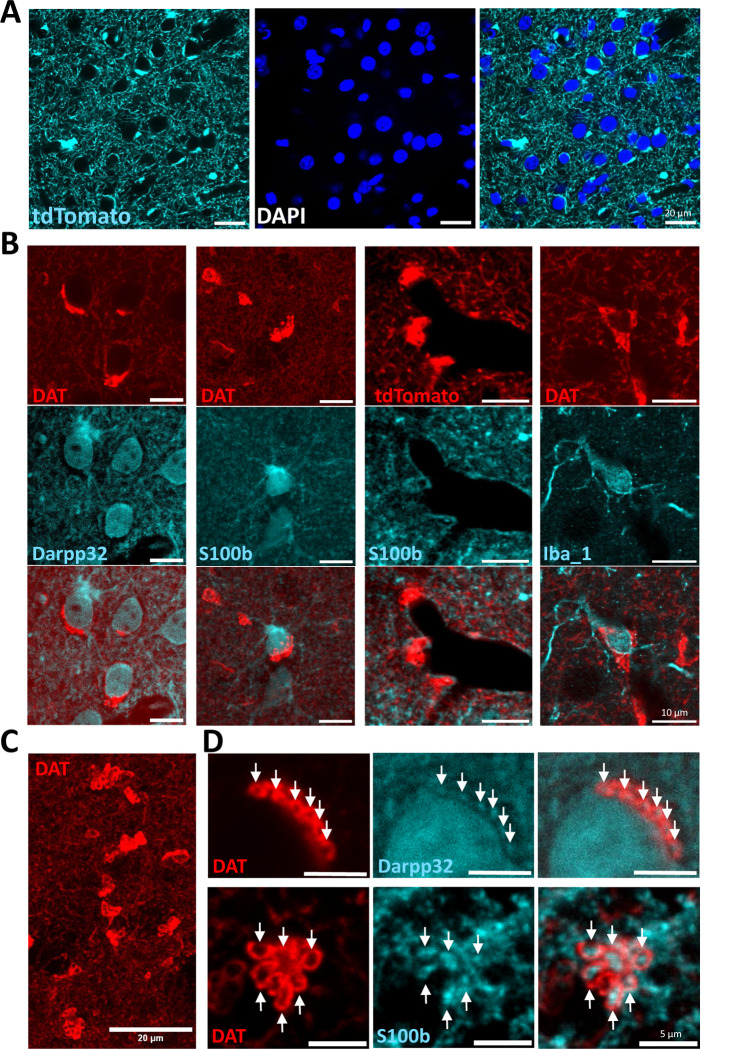
Dystrophic axon terminals of DAergic Synj1^RQ^ neurons are generally localized next to cell bodies and blood vessels. **A**. Juxtaposition of tdTomato-positive dystrophic axon terminals to DAPI-positive cell bodies. **B**. Double immunofluorescence for DAT and for markers of different cell populations: Darpp32 (marker of medium spiny neurons), S100b (marker of astrocytes), Iba1 (marker of microglia). **C**. Abnormal foci of DAT immunoreactivity are represented by clusters of rings. **D**. Double immunofluorescence for DAT and for DARPP32 or for S100b shows that dystrophic axon contains evaginations of the neighboring cell. Scale bars: **A**, 20 μm; **B**, 10 μm; **C**, 20 μm; **D**, 5 μm. **A**: 3 month-old male; **B**-**D**: 3–5 month-old females and males.

Dystrophic tdTomato- and DAT-positive axonal foci in the DLS were generally adjacent to cell bodies and blood vessels (Fig. 3A and 3B). These included cell bodies of spiny projection neurons (SPNs) (DARPP32 immunoreactive) (Nishi et al. 1997), of astrocytes (S100b immunoreactive) (Ludwin et al. 1976; Du et al. 2021) and of microglia (Iba1 immunoreactive) (Imai et al. 1996), thus ruling out specificity for a given cell type (Fig. 3B). Moreover, high power views of DAT immunoreactivity revealed that abnormal foci were represented by clusters of rings (Fig. 3C and D) whose core was positive for markers of the adjacent cell (Fig. 3D), indicating a tight interdigitation of these axonal structures with the neighboring cell.

Inspection at high resolution of the tdTomato fluorescence of 3D volumes of delipidated DLS revealed occasional dystrophic axons that made contacts with multiple cell bodies (Fig. S2). However, generally, dystrophic axonal foci did not appear to be interconnected suggesting that they reflect local changes rather than disruption of an entire axonal arbor.

### Dystrophic foci correspond to membrane whorls often surrounding evaginations of the adjacent cell.

**Fig. 4.**
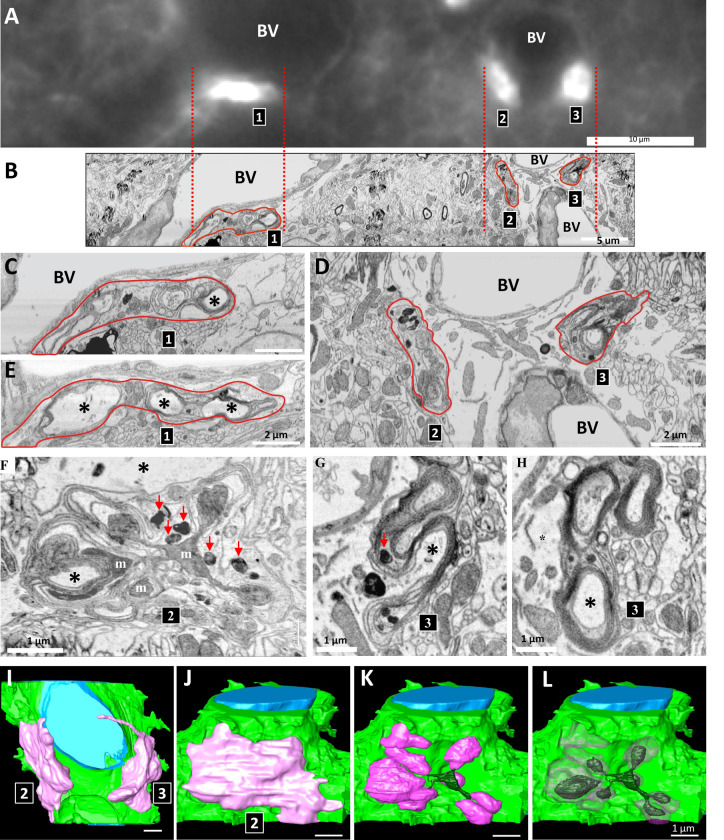
CLEM-FIB-SEM analysis of tdTomato expressing dystrophic DAergic axons in the striatum of a Synj1^RQ^ mouse (4 month-old female). **A** and **B**. tdTomato fluorescence (top) and FIB-SEM image of the same region of the striatum at the same magnification. The FIB-SEM image (**B**) shows that the three clusters (numbered 1,2 and 3) of tdTomato fluorescence visible in (**A**), correspond to multilayered membrane structures. **C** and **D**. Same FIB-SEM images as in **B** but at higher magnification. **E**. Another view of cluster 1 but at a different level in the 3D volume. **F**-**H**. Different views of clusters 2 and 3 at different levels within the 3D volume and at higher magnification relative to field **D**. Black asterisks in fields **C** and **E**-**H** indicate evaginations of astrocytes engulfed by dystrophic axons (see also movie 2). m = mitochondria. **I** and **J**. 3D surface view of the dystrophic axons forming clusters 2 and 3. Green = surface of an astrocyte; blue = blood vessel lumen; pink = surface view of the dystrophic terminal. **K** and **L**. Reconstruction of membranes within the dystrophic terminal. magenta = membrane whorls, dark green = mitochondria. The images of this figure were generated from movie S2 and S3. Scale bars: **A**, 10 μm; **B**, 5 μm; **C**-**E**, 2 μm; **F**-**L**, 1 μm.

To unambiguously determine the ultrastructure of dystrophic axonal foci, we performed correlative light electron microscopy (CLEM). In order to precisely identify the tdTomato fluorescent structures of interest we used for EM a 3D technique: Focused Ion Beam Scanning Electron Microscopy (FIB-SEM) (Wu et al. 2017; Xu et al. 2021). Vibrotome sections (50 μm thick) were first observed by fluorescence microscopy to define regions of interest. Sections were then embedded in epon, and epon blocks where processed for FIB-SEM initially using a commercial Zeiss crossbeam 550 machine. 3D EM volumes were subsequently aligned with fluorescence images, revealing that foci of tdTomato fluorescence corresponded to clusters of membrane whorls or onion-like structures as illustrated in Fig. 4 and movies S4 and S5 which show two such elements close to a blood vessel. Within a cluster, these elements were interconnected, indicating that they originate from the same axon. This was also clearly exemplified by the 3D reconstruction of a very large branched mitochondrion in one of the foci shown in Fig. 4F, where such branches extended into different membrane whorls within the same cluster (Fig. 4K and L). The cores of these “onions” often harbored evaginations of nearby cells, consistent with light microscopy observations (asterisks in Fig. 4C–H).

### Membrane whorls derive from massive invaginations of the plasma membrane.

**Fig. 5.**
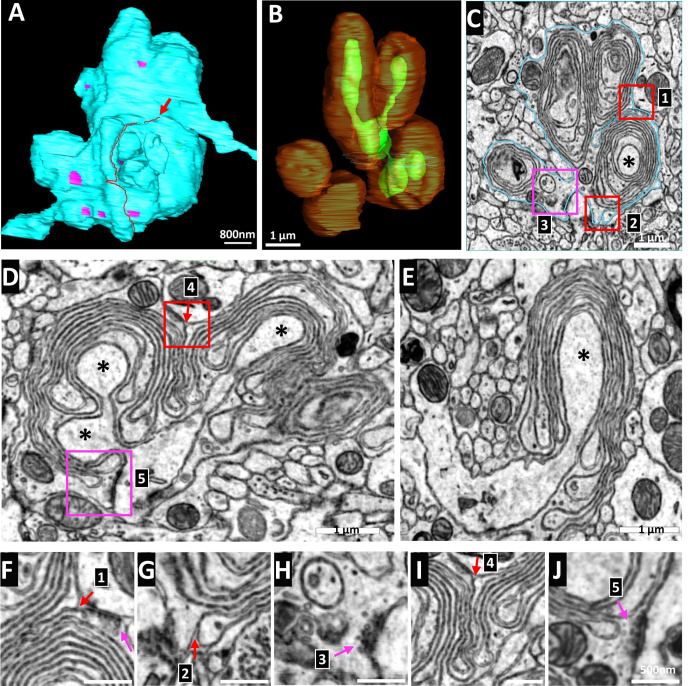
Analysis of a branched dystrophic nerve terminal of Synj1^RQ^ striatum with a customized high resolution FIB-SEM device (8 month-old female). **A** and **B**. 3D reconstruction of a dystrophic nerve terminal. **A** shows a surface view, with a red line (arrows) indicating where the outer plasma membrane folds inwards to generate the lamellar onion-like structures. **B** shows the onion-like structures (brown) which enclose evaginations (green) of an adjacent neuron. A synapse of the dystrophic terminal onto the neuron is visible in square #5 shown at high magnification in field J. **C** - **E** Single FIB-SEM slices of the 3D volume of the dystrophic nerve terminal shown in **A** and **B**. A blue line in **C** outlines the outer plasma membrane. Asterisks mark evaginations of an adjacent neuron. **F** - **J**. High magnification views of the numbered rectangles in **C** and **D**. Arrows in **F**, **G** and **I** point to where the outer cell plasma membrane invaginates to generate the massive onion-like whorls. Arrows in H and J point to two small synaptic vesicle clusters at active zones where the dystrophic nerve terminal makes synaptic contacts with a neighboring cell. Scale bars: **A**, 800 nm; **B**-**E**, 1 μm; **F**-**J**, 500 nm.

Higher resolution views of Synj1^RQ^ mutant striata were obtained by a customized FIB-SEM machine at Janelia Research Campus, as shown in Fig. 5 (Xu et al. 2017; Pang and Xu 2023). The dystrophic axon shown in this figure comprises multiple whorls that enwrap evaginations of a neighboring neuron (Fig. 5B, D and E; movie S6 and S7).

**Fig. 6.**
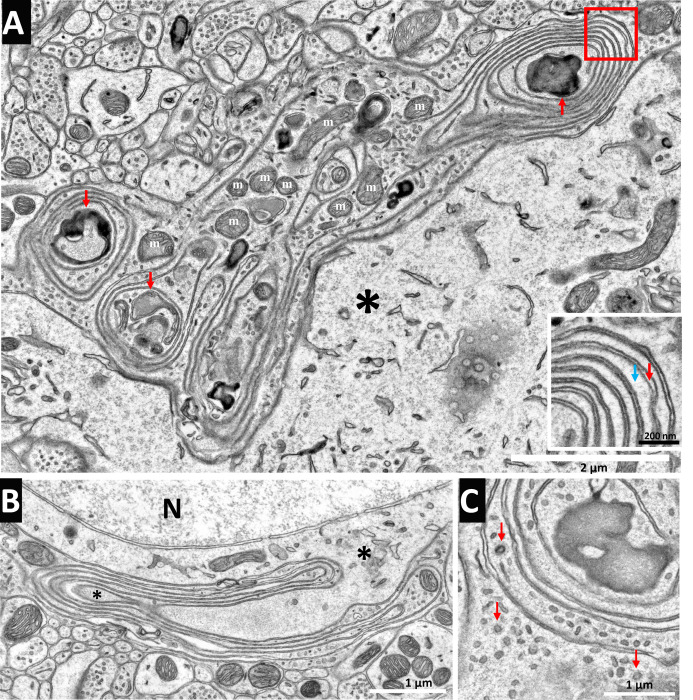
Conventional TEM demonstrating presence of trapped organelles in the membrane whorls (1.5 month-old male). **A**. Example of lamellar plasma membrane invaginations of a dystrophic axon including a variety of cell organelles among individual lamellae. Dense organelles (red arrows), possibly degenerating mitochondria or lysosomes, are present in the core of onion-like whorls. An asterisk indicates a neighboring cell. M = mitochondria; SV = synaptic vesicles. Inset: high magnification view of the region enclosed by a red square in the main field; red and blue arrows point to extracellular space and cytosolic space respectively. **B**. Multilayered plasma membrane invagination enclosing the evagination of a neighboring cell (asterisk). **C**. SVs and three clathrin coated vesicles are visible among lamellae. Scale bars: **A**, 2 μm, inset, 200 nm; **B** and **C**, 1 μm.

Close inspection of the multilayered membrane structures in FIB-SEM volumes (see arrows in fig.5 C, D, F, G and I) allowed to identify them as sheet-like invaginations of the axonal plasma membrane that wrap around portions of the adjacent cell (Fig. 5D and E). While these images are reminiscent of myelin, in this case it is the plasma membrane of the DA axons that forms these invaginations, as discussed previously (Cao et al. 2017), and not the plasma membrane of oligodendrocytes. This was consistent with the intense immunoreactivity of membrane whorls for DAT (Fig. 1C, 3C and 3D), a marker of the axonal plasma membrane of DA neurons. The shape of these structures explains why large foci of DAT immunoreactivity have a ring-like shape when observed at high power fluorescence microscopy (see Fig. 3C and 3D). Further confirming the axonal identity of these structures was the continuity with presynaptic active zones (Fig. 5F, H and J) and the presence scattered synaptic vesicles among lamellae, as it can be seen more clearly in specimens analyzed by conventional TEM (Fig. 6).

In some cases, the core of onion-like structures contained organelles with an electron-dense appearance (Fig. 4F and 6A). At least some of them appeared to be degenerating mitochondria, other may be lysosomes or autophagosomes. Possibly, organelles trapped in these structures, either in the dystrophic terminals, or in the evaginations of the cells that they enwrap, undergo degenerative changes due to their partial segregation from other portions of the cell.

### Synaptic vesicle markers of DAergic neurons are not enriched in the membrane whorls.

**Fig. 7.**
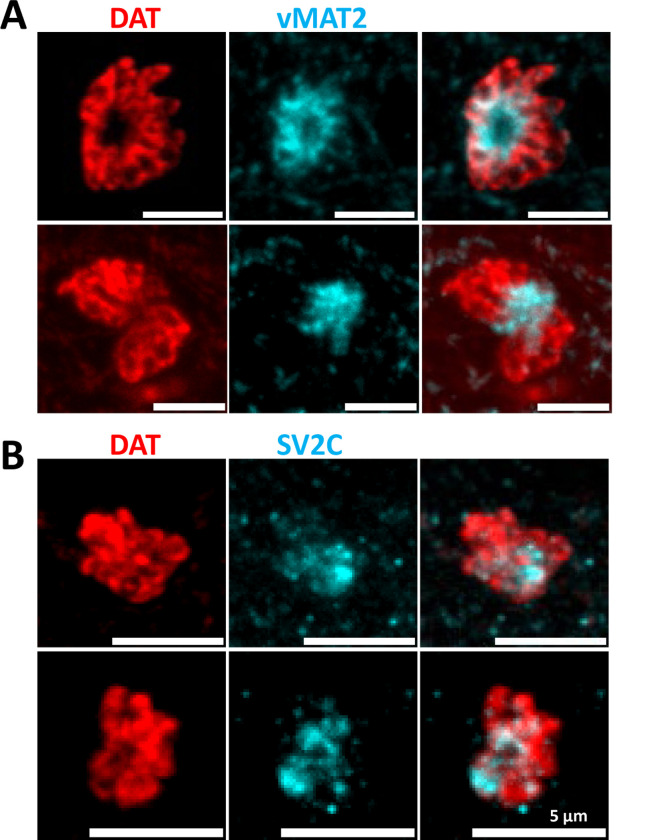
Segregation of DAT from markers of dopamine containing synaptic vesicle proteins in dystrophic nerve terminals. **A** and **B**. Double immunofluorescence for DAT and the synaptic vesicle membrane proteins vMAT2 and SV2C. The different localization of the two vesicular markers relative to DAT within the dystrophic axonal foci indicate that the membrane invaginations are not enriched in synaptic vesicle proteins. Scale bars: 5 μm. (4–5 month-old males and females).

As Synj1 is implicated in the endocytic recycling of synaptic vesicles (Cremona et al. 1999; Kim et al. 2002; Mani et al. 2007; Milosevic et al. 2011), it was of interest to determine whether vesicle proteins known to be enriched in synaptic vesicles of DAergic nerve terminals, SV2C (Janz and Südhof 1999; Dunn et al. 2017) and vMAT2 (Onoa et al. 2010), were enriched in the whorls. SV2C and vMAT2 immunoreactivity were concentrated in abnormal axonal foci, but did not overlap with DAT, indicating that formation of the massive plasma membrane invagination is a process distinct from the endocytic events that recapture synaptic vesicle proteins after their exocytosis (Fig. 7A and B).

## DISCUSSION

Animal models of disease are powerful experimental tools to dissect disease mechanisms. While more than 20 mutations responsible for familial forms of PD have already been identified (Blauwendraat et al. 2020; Cogan et al. 2025) in most cases the introduction of the corresponding mutations in mice does not result in an obvious disease phenotype. Mice with the R258Q patient mutation in Synj1 have at least some of the neurological phenotypes observed in human patients (Cao et al. 2017; Ng et al. 2023). Importantly, dystrophic changes in a subpopulation of DAergic axons are present in the striata of these mice and these changes are largely restricted to the dorsolateral striatal regions known to be primarily involved in the motor dysfunction of PD patients(Hilário and Costa 2008; Fushiki et al. 2024; Mantas et al. 2025). As we have shown, these changes are accompanied by a defect in evoked DA release specifically in the same striatal region. Thus, studies of these mice may help shed light on the special vulnerability of these neurons.

Here we have further elucidated the nature of these focal dystrophic changes. Using FIB-SEM CLEM we have proven that they are represented by massive invaginations of the axonal plasma membrane that form onion-like structures somewhat reminiscent of myelin. However, besides the difference of the cell involved (neurons rather than oligodendrocytes), the whorls represent invaginations, rather than evaginations of the plasma membrane as in myelin. Moreover, in contrast to the layers of myelin, where membranes are tightly apposed with no intervening cytoplasm, lamellae of these axonal invaginations contain thin layers of cytoplasm and in some cases also small organelles. As the number of dystrophic axonal segments is observed in a minor subpopulation of the DA axons even in striatal regions where they are most abundant, it remains unclear whether many axons of few neurons, or few axonal branches of different neurons, are affected. We have further shown that these onion-like structures trap evaginations of adjacent cell bodies, raising the possibility of cell non-autonomous effects in the pathological manifestations. Much remains to be learned about mechanisms underlying these striking pathological changes.

DAT is a major component of these whorls. In contrast, synaptic vesicle proteins known to be enriched in vesicles of DA nerve terminals, such as SV2C and vMAT2 (Janz and Südhof 1999; Onoa et al. 2010; Dunn et al. 2017), do not colocalize with DAT-positive membrane whorls although they are enriched in these dystrophic axonal segments. Possibly they reflect synaptic vesicles trapped in the axon dilations. This speaks against the possibility that the dramatic expansion of the plasma membrane responsible for the formation of the membrane invaginations may simply reflect synaptic vesicle exocytosis not balanced by synaptic vesicle endocytosis. In fact, exocytosis of synaptic vesicles may be impaired in mutant terminals, as supported by the defect in evoked DA release detected by our functional experiments. A selective stranding of DAT at the plasma membrane delivered to the plasma membrane by some form of constitutive exocytosis, must be involved.

Patients with loss of function mutations in auxilin (alias: DNAJC6 or PARK19) develop early onset parkinsonism with clinical manifestations very similar to those of patient with the SYNJ1^RQ^ mutation (Edvardson et al. 2012; Köroğlu et al. 2013; Krebs et al. 2013; Quadri et al. 2013). Interestingly, dystrophic changes in DAergic axons very similar - in both morphology, localization and enrichment in DAT - to those observed in the DLS of Synj1^RQ^ are present in the DLS of auxilin KO mice (Ng et al. 2023; Vidyadhara et al. 2023). Interestingly, auxilin and Synj1 are functional partners in endocytosis (Saheki and De Camilli 2012). The PI(4,5)P_2_ phosphatase activity of Synj1 allows release of clathrin adaptors and other endocytic factors anchored to the membrane via an interaction with PI(4,5)P_2_ (Cremona et al. 1999; Verstreken et al. 2003; Milosevic et al. 2011). Auxilin, a cofactor of the ATPase HSC70, is implicated in clathrin coat disassembly (Eisenberg and Greene 2007; He et al. 2020). In agreement with the functional partnership of these two proteins with house-keeping functions in neurons, a decreased number of synaptic vesicles and an increased number of clathrin coated vesicles is observed at synapses throughout the brain, including at striatal synapses, of mice harboring Synj1 or auxilin loss-of-function mutations (Cremona et al. 1999; Yim et al. 2010; Cao et al. 2017; Ng et al. 2023; Vidyadhara et al. 2023). The changes observed in dystrophic DAergic neurons, however, do not represent an exaggeration of this phenotype. In fact, only occasional clathrin coated vesicles are observed in proximity of the membrane whorls. Perhaps, as mentioned above, the onset of the dystrophic changes correlates with a shutdown of SV exocytosis in these terminals.

Recently, it was shown that abnormal dystrophic swelling of DAergic axons occurs also in the ventral striatum of mice in which the Synj1^RQ^ mutation is combined with the loss of auxilin (Ng et al. 2023), or in mice with conditional KO of Synj1 in DA neurons rather than simply harboring the Synj1^RQ^ mutation which selectively impair the Sac1 phosphatase activity of this protein (Lin et al. 2026). Thus, it would appear that the axonal arbors of DA neurons are particularly vulnerable to the lack of Synj1 and auxilin function, with more severe vulnerability of the subpopulation projecting to the DLS.

It was reported that Synj1 haploinsufficiency results in increased levels of DAT in DAergic neurons, but in reduced surface expression of DAT (Saenz et al. 2024). This would seem in contrast with our evidence that in mice with impaired Synj1 function a massive amount of DAT is trapped in the dramatically expanded plasma membrane of a subset of axons. However, while the mutation of Synj1^RQ^ mutant mice abolishes its 4-phosphatase activity (Krebs et al. 2013; Quadri et al. 2013), reduced surface expression of DAT in mice with a *Synj1* KO allele was attributed to impaired 5-phosphatase activity (Saenz et al. 2024). The trapping of DAT at the plasma membrane is also supported by the reports that in auxilin mutant mice, where similar DAT-enriched structures are observed, such structures are accessible to a membrane impermeant DAT ligand (Vidyadhara et al. 2023).

A key open question is why the dystrophic changes described here in Synj1^RQ^ mice occur so specifically in a subpopulation of DAergic axons of the DLS. Our functional studies of evoked DA release in striatal slices have revealed a defect of such release in the same brain region and not in the ventral striatum. It is unlikely, however, that such defect may be accounted for by the relatively small percentage of dystrophic axons. We favor a model in which the Synj1^RQ^ mutation results in a general selective impairment of DA neurons projecting to the dorsal striatum. In a subset of axonal segments this functional impairment may reach a threshold beyond which the normal balance between exocytosis and endocytic mechanisms, including constitutive exocytosis-endocytosis to maintain plasma membrane homeostasis, may be chronically perturbed, leading to massive plasma membrane invaginations and synaptic vesicle exocytosis shut down. Elucidating the precise causes for the selective vulnerability of DAergic neurons to the lack of Synj1 and auxilin, and more so of the subset of neurons projecting to the DLS, remains an important priority for future studies.

## METHODS

### Antibodies.

Rabbit anti-DARPP-32 (2306, RRID: AB_823479) from Cell Signaling Technology; rat anti-DAT (MAB369, RRID: AB_2190413) from Millipore;; rabbit anti-SV2C (119203, RRID: AB_993023), from Synaptic Systems; mouse anti-S100b (SAB4200671, RRID: AB_3712829) from Sigma; rabbit anti-Iba1 (01919741, RRID: 839504) from FUJIFILM Wako Pure Chemical Corporation; rabbit anti-vMAT2 (MSFR107570, RRID: AB_2571857) from FRONTIER INSTITUE CO., LTD.

### Mice.

The following mouse strains were interbred to generate mice expressing tdTomato in DAergic neurons and harboring either the WT or R259Q *Synj1* allele: DAT^IREScre^ (B6.SJ-LSlc6a3^tm1.1(cre)Bkmn^/J; RRID: IMSR_JAX-006660); Ai9 mice (B6.Cg-Gt(ROSA)26Sor^tm9(CAG-tdTomato)Hze^/J; RRID: IMSR_JAX-007909); Synj1^RQ^ mice in C57BL6 (C57BL/6-*Synj1*<R259Q>); RRID: MGI:6120537 (Cao et al. 2017). Mice heterozygous for DAT^IREScre^ and either homozygous or heterozygous for the conditional tdTomato cassette were used for our experiments. All mice were maintained on a 12 h light/dark cycle with standard mouse chow and water ad libitum. All research and animal care procedures were approved by the Yale University Institutional Animal Care and Use Committee.

### Brain Immunofluorescence.

Mice were anesthetized with a Ketamine (VetOne, NDC 13985–584-10)/Xylazine (Anased, NDC 59399–110-20) anesthetic cocktail injection, perfused transcardially with 37 °C pre-warmed 4% PFA and 0.05% Glutaraldehyde (Electron Microscopy Sciences) in 0.1M phosphate buffer (PB), PH7.4 and the brains were kept in the same fixative overnight at 4 °C. Brains were then washed 3 times for 20 min in 0.1M PB buffer and 25–30 μm thick coronal or sagittal sections were cut with a vibratome. Floating sections were incubated in 2% BSA, either 2% donkey (EMD Millipore, S30–100ML) or goat (ThermoFisher Scientific, 16210064) serum, 0.4% Triton X-100, 0.05% Tween20, 0.01% NaN_3_, and 50mM NH_4_Cl in 0.1M PB buffer for 2 h at room temperature and then incubated with primary antibodies (diluted in the same buffer) overnight at 4 °C. Subsequently, sections were washed 3 times for 10 min with 0.1M PB buffer, then incubated with Alexa-conjugated secondary antibodies for 2 h at room temperature. Finally, sections were mounted with ProLong^™^Gold antifade reagent (Invitrogen, P36934) and sealed with nail polish. Images were acquired either with an Olympus slice view VS200 slide scanner equipped with a Hamamatsu Orca-Fusion camera and a 20x Olympus UPlanXApo objective or a Nikon Ti2-E inverted microscope (Yokogawa CSU-W1 SoRa, Nikon) equipped with a 60x SR Plan Apo IR oil-immersion objective. Images analysis was done with Fiji software. A detailed protocol is found at DOI: https://dx.doi.org/10.17504/protocols.io.14egn515zg5d/v1.

### Brain tissue clearing and imaging.

Brain tissue clearing was performed following the standard SHIELD protocol and passive delipidation procedure (LifeCanvas). Briefly, mice were perfused with 4% paraformaldehyde (PFA) and 0.05% glutaraldehyde in 0.1 M phosphate buffer (PB) as described above, and brains were post-fixed overnight in the same fixative at 4 °C. Tissues were then crosslinked by incubation in 20 mL SHIELD OFF solution for 3 days at 4 °C, followed by 20 mL SHIELD ON solution for 24 h at 37 °C with shaking (40 rpm, orbital shaker). Subsequently, brains were transferred to 20 mL delipidation buffer for 1 week at 45 °C with shaking (60 rpm). After delipidation, tissues were incubated under shaking (60 rpm) in 20 mL of 50% EasyIndex solution (RI = 1.52) in H_2_O for 24 h at 37 °C, followed by 100% EasyIndex at 37 °C for an additional 24 h until fully transparent.

#### Full brain imaging by a light-sheet microscope.

The cleared mouse brain was embedded in an index-matching gel block and immersed in an imaging chamber filled with immersion oil (refractive index (RI) 1.52). Imaging was performed using a SmartSPIM light-sheet microscope (LifeCanvas Technologies) equipped with a 9×, 0.3 NA clearing objective (Applied Scientific Instrumentation). A total of 4 × 6 tiles were acquired and stitched together using the manufacturer’s post-acquisition software. The left striatum was segmented and the neighboring areas were masked out based on tdTomato fluorescence intensity. The striatum fluorescence dataset was then preprocessed to remove background and nonspecific signal, followed by threshold-based segmentation of tdTomato-positive puncta corresponding to dystrophic dopaminergic axons using Zeiss Arivis Pro.

#### Confocal imaging of vibratome sections.

For high-resolution confocal imaging, brains were washed in 0.1 M PB for 6 h with one buffer change. Sagittal brain sections (100 μm thick) were cut using a vibratome and re-incubated in EasyIndex for at least 20 min until transparency was restored. Sections were mounted on glass slides in one drop of EasyIndex Matched Immersion Oil (RI = 1.52), cover-slipped (12 mm), and imaged using a Nikon Ti2-E equipped with a Yokogawa CSU-W1 SoRa (Nikon) and a 60× SR Plan Apo IR oil-immersion objective (IMMERSION OIL TYPE F, RI = 1.518). 3D image stacks (200 × 200 × 100 μm^3^) were acquired at high spatial resolution (108 × 108 nm per pixel in XY, 100 nm z-step), enabling detailed visualization of fine structures. Image datasets were subsequently processed and analyzed using Imaris (Oxford Instruments) and Dragonfly Pro (Comet Technologies Canada Inc.) software.

Details of the protocol can be found at: https://sites.google.com/lifecanvastech.com/protocol/outline

### FIB-SEM CLEM at the Yale CCMI facility.

Mouse anesthesia and transcardial perfusion with fixative (4% paraformaldehyde and 0.05% glutaraldehyde in 0.1M PB buffer), followed by an overnight incubation with the same fixative and subsequent washes, were performed as described above. Sagittal sections (50 μm) were then prepared using a vibratome. Sections were mounted on glass slides in 0.1 M PB, cover-slipped, and fluorescence images were acquired using an Olympus SliceView VS200 slide scanner equipped with a Hamamatsu Orca-Fusion camera and a 40× Olympus UPlanXApo objective. Sections including striatal regions enriched in clusters of tdTomato fluorescence were selected for further FIB-SEM processing and processed in sequence through the following steps: incubation in 2.5% glutaraldehyde, 2 mM CaCl_2_ (J.T. Baker) in 0.1 M sodium cacodylate buffer for 2–3 hours on ice; incubation in 2% osmium tetroxide, 1.5% potassium ferrocyanide (K_4_Fe(CN)_6_) (Sigma-Aldrich), and 2 mM CaCl_2_ in 0.1 M sodium cacodylate buffer for 1 hour on ice; incubation with thiocarbohydrazide (TCH) for 20 minutes at room temperature; a second incubation in 2% osmium tetroxide in water for 30 minutes at room temperature; incubation in 2% aqueous uranyl acetate overnight at 4 °C; dehydration through a graded ethanol series (20%, 50%, 70%, and 90%; 5 minutes each), followed by three changes of 100% ethanol for 5 minutes each. Sections were then transferred to propylene oxide for 10 minutes at room temperature and infiltrated with Epon resin (Embed 812) in propylene oxide at 25% (several hours), 50% (several hours), and 75% (overnight), followed by 100% Epon overnight and fresh 100% resin for several additional hours. Finally, sections were mounted onto 12-mm glass coverslips in a drop of Epon, excess resin was removed, and samples were transferred to 60 °C oven for 48 hours to induce epon polymerization.

Coverslips with Epon-embedded brain sections were glued onto scanning electron microscope (SEM) aluminum sample-mounting stubs, and platinum *en bloc* coating on the sample surface was carried out with a sputter coater (Ted Pella, Inc.). SEM imaging of the surface of the sections was performed to define regions to be analyzed with FIB-SEM by aligning major tissue landmarks, such as blood vessels, cell bodies and axon bundles with the same structures as observed by previous fluorescence microscopy of the same sections in areas enriched in dystrophic DAergic axon (large clusters of tdTomato fluorescence. FIB-SEM imaging of these regions was performed using a Crossbeam 550 FIB-SEM workstation operating with SmartSEM (Carl Zeiss Microscopy GmbH) and the Atlas 5 engine (Fibics Incorporated). The imaging resolution was set at 8 nm/pixel in the x, y axis with milling being performed at 4 nm/step along the z axis (binned down by 2 when images were exported) to achieve an isotropic resolution of 8 nm/voxel. Images were aligned and exported with Atlas 5 (Fibics Incorporated), further processed, and 3D reconstructed with DragonFly Pro software (Comet Technologies Canada Inc). Except when noted, all reagents were from Electron Microscopy Sciences. A detailed protocol is found at: https://dx.doi.org/10.17504/protocols.io.5jyl84×49g2w/v1.

### FIBSEM at HHMI Janelia Research Campus.

Epon-embedded samples (less than 1 mm in all dimensions) were mounted on sample studs and their tops were trimmed to a 40–100- × 100–300-nm dimension. Samples were then re-embedded in durcupan (EMS, Catalog #14040) by dabbing gently on the trimmed top of the epon-embedded sample with a small volume of uncured durcupan and then exposing them to 60 °C. Next, cured samples were coated with a thin layer of 10-nm gold and 100-nm carbon. For FIB-SEM imaging, a custom built FIB-SEM, with an FEI Magnum FIB column mounted onto a Zeiss Merlin SEM, was used. SEM imaging was performed using a 200-pA electron beam of 700 V landing energy at 200 kHz with 2-nm spacing in the *x* and *y* directions. A 7-nA 30-kV gallium ion beam was used to remove 2-nm of material in the *z* axis after each SEM image to generate an 8-nm isotropic voxel image stack by an InLens detector (Zeiss electron microscope) in which secondary electrons were filtered out through sample biasing so that only backscattered electrons were detected. For the generation of the 3D reconstructions, membrane contours were traced semi-manually by using 3dmod software. A detailed procedure is available at: https://doi.org/10.25378/janelia.24222688 and in Pang et al. 2023 (Pang and Xu 2023). See also (Xu et al. 2017).

### Conventional TEM imaging.

DLS regions of Epon-embedded brain tissue sections prepared as described above for FIBSEM were cut with a Leica ultramicrotome to generate 60 nm ultrathin sections. The sections were post-stained with uranyl acetate substitute (Uranyless, EMS), followed by a lead citrate (EMS) solution. Sections were observed in a Talos L 120 C TEM microscope at 80 kV and images were taken with Velox software and a 4k × 4 K Ceta CMOS Camera (Thermo Fisher Scientific). A detailed procedure is available at: https://dx.doi.org/10.17504/protocols.io.36wgqxx65lk5/v1.

### Ex vivo slice preparation for the analysis of DA release.

Mice were terminally anesthetized with a mixture of ketamine (50 mg/kg) and xylazine (4.5 mg/kg) and transcardially perfused with ice-cold modified artificial cerebrospinal fluid (aCSF) (“slicing solution,” containing 49.14 mM NaCl, 2.5 mM KCl, 1.43 mM NaH_2_PO_4_, 25 mM NaHCO_3_, 25 mM glucose, 99.32 mM sucrose, 10 mM MgCl_2_, and 0.5 mM CaCl_2_); after decapitation, the brain was removed and sectioned in 220-μm-thick coronal slices using a vibratome (VT1200S, Leica Microsystems) in the same slicing solution. Slices were transferred into a holding chamber containing recording aCSF (containing 135.7 mM NaCl, 2.5 mM KCl, 1.25 mM NaH_2_PO_4_, 25 mM NaHCO_3_, 2 mM CaCl_2_, 1 mM MgCl_2_, and 3.5 mM glucose). Slices were allowed to recover for 30 min at 34°C and then kept at room temperature for at least 15 min before starting the experiments. All solutions were pH 7.4 and ~310 mOsm and were continually bubbled with 95% O_2_ and 5% CO_2_. A detailed procedure is available at: https://dx.doi.org/10.17504/protocols.io.ewov1rb8ylr2/v1.

### Two-photon laser scanning microscope (2PLSM) workstation.

The 2PLSM workstation used was an Ultima dual-excitation-channel scan head (Bruker Nano Fluorescence Microscopy Unit). The foundation of the system is the Olympus BX-51WIF upright microscope with an LUMPFL 60×/1.0 NA (numerical aperture) water-dipping objective lens. The automation of the XY stage motion, lens focus, and manipulator XYZ movement was provided by an FM-380 shifting stage, an axial focus module for Olympus scopes, and manipulators (Luigs & Neumann). The 2P excitation (2PE) source was a Chameleon Ultra1 series tunable wavelength (690 to 1040 mm, 80 MHz, ~250 fs at sample) Ti: sapphire laser system (Coherent Laser Group). Each imaging laser output is shared (equal power to both sides) between two optical workstations on a single anti-vibration table (TMC). Workstation laser power attenuation was achieved with two Pockels’ cell electro-optic modulators (models M350–80-02-BK and M350–50-02-BK, Con Optics) controlled by Prairie View 5.6 software. The two modulators were aligned in series to provide enhanced modulation range for fine control of the excitation dose (0.1% steps over 5 decades), to limit the sample maximum power. The fluorescence emission was collected by non–de-scanned photomultiplier tubes (PMTs). Green channel (490 to 560 nm) signals were detected by a Hamamatsu H7422P-40 select GaAsP PMT. Red channel (580 to 630 nm) signals were detected by a Hamamatsu R3982 side on PMT. Dodt-tube–based transmission detector with Hamamatsu R3982 side on PMT (Bruker Nano Fluorescence) allowed visualization of slices during laser scanning. Scanning signals were sent and received by the National Instruments PCI-6110 analog-to-digital converter card in the system computer (Bruker Nano Fluorescence). All spiral images were collected with a pixel size of 0.393 μm, a pixel dwell time of 8 μs, and a frame rate of 39.165 fps (frames per second). A detailed protocol is found at: https://dx.doi.org/10.17504/protocols.io.bp2l6jwn1vqe/v1.

### DA release measurements.

Mice homozygous or heterozygous for the RQ mutation, referred to as Synj1^RQ^ mice and *Synj1*^*+/RQ*^ or control mice, respectively, were stereotaxically injected with 500 nl of AAV5-CAG-dLight1.3b Addgene #125560) into either the DLS (AP: 0.3, ML: 1.3, DV: 3.1) or NAc (AP: 1.1, ML: 1.1, DV 4.0) at P30. The Allen Mouse Brain Atlas, online version 1, 2008 (http://mouse.brain-map.org) was the source of all stereotaxic coordinates. Experiments were performed at P45 (+/− 2 days). Mice were anesthetized, perfused and brain slices prepared as described above and previously (Zampese et al. 2022). dLight imaging was performed with 920 nm light using the 2PLSM system, as defined above. dLight samples were imaged with 20 μm diameter field of view (continuous spiral scan with 95% duty cycle using 84,267 px, 8.0 μs pixel dwell time at 39.165 f.p.s. All ROI signals were summed per frame. The spiral scan data (outside of the circle towards the inside of the circle) has 3000 ms of pre-stimulation baseline (*f*_0_, with laser blocked background counts subtracted). DA release was evoked by electrical stimulation (600 μA, 0.2 ms pulse width, 1P or 5 pulses at 10 Hz) using a tungsten bipolar electrode inserted approximately 30 μm to either the DLS or ventral striatum. In some experiments a stimulation was applied every 10 seconds for 5 times (Virmani et al. 2006; Platt et al. 2012). Boxplots quantifying DA release are presented as a percentage of dF max (attained by applying 100 μM DA to the slice). The time-series plots display Δ*f*/*f*_0_ normalized to the first response in the series. Traces representing DA release are normalized to ΔF max to allow comparisons between slices and animals. A detailed protocol is found at: https://dx.doi.org/10.17504/protocols.io.bp2l6jwn1vqe/v1.

### Analysis and statistics.

Images were analyzed offline using FIJI (Schindelin et al. 2012). Traces were subsequently analyzed using Excel (Microsoft) and analyzed in GraphPad Prism8 (GraphPad software). Sample *n* represents the number of brain slices collected from *N* animals. Statistical analysis was performed using GraphPad Prism8. The non-parametric Mann-Whitney U test was used to assess statistical significance in experiments measuring absolute DA release. For experiments investigating vesicle recycling, a two-way repeated measures ANOVA was used in conjunction with the Šídák method to correct for multiple comparisons. Probability threshold for statistical significance was P < 0.05.

## Supplementary Material

Supplement 1

Supplement 2

Supplement 3

Supplement 4

Supplement 5

Supplement 6

Supplement 7

Supplement 8

Supplement 9

Supplement 10

Supplement 11

## Data Availability

The data, protocols, and key lab materials used and generated in this study are listed in a Key Resource Table alongside their persistent identifiers at [https://doi.org/10.5281/zenodo.20769706]. No code was generated for this study; all data cleaning, preprocessing, analysis, and visualization was performed using ImageJ, Zeiss Arivis Pro, Dragonfly Pro (Comet Technologies Canada Inc), Imaris, OLYMPUS OlyVIA 4.2, Atlas 5 (Fibics Incorporated), Velox (Thermo Fisher Scientific), 3dmod, Handbreak, Excel and GraphPad Prism8.
