# Dystrophic changes of nigrostriatal axons harboring a Synj1 Parkinson mutation suggest catastrophic failure of endocytic mechanisms

**DOI:** 10.64898/2026.06.24.733515

**Published:** 2026-09-10

**Authors:** Yumei Wu, Peng Xu, James Moran, C. Shan Xu, Kenneth J. Hayworth, Mian Cao, Lin Shao, D. James Surmeier, Harald F. Hess, Pietro De Camilli

## Abstract

Synaptojanin 1 is a brain enriched phosphoinositide phosphatase implicated in endocytosis at the synapse. A mutation (R258Q) that selectively impairs its Sac1 phosphatase domain causes early onset familial Parkinsonism. Neurons of mice with this mutation display synaptic vesicle traffic defects across the brain, but selective dystrophic changes in a subset of dopaminergic axons in the dorsolateral striatum. Using correlative light microscopy-FIB-SEM of mutant mouse striata to visualize in 3D these abnormal structures we show that they represent clusters of focal axonal dilations harboring massive, onion-like DAT enriched plasma membrane infoldings, generally localized next to cell bodies of neighboring cells, often engulfing evaginations of such cells. This dysmorphia was associated with a deficit in dopamine release in the same striatal region. Given the involvement of Synj1 in endocytic mechanisms, these structures may reflect an imbalance between exocytosis and endocytosis. Their occurrence only in a subset of axons suggest a vulnerability threshold of these axons beyond which the expansion of the plasma membrane is not counteracted by compensatory mechanisms.

## Full Text Availability

The license terms selected by the author(s) for this preprint version do not permit archiving in PMC. The full text is available from the preprint server.

