# Supplementary material for: Dystrophic changes of nigrostriatal axons harboring a Synj1 Parkinson mutation suggest catastrophic failure of endocytic mechanisms
Source: bioRxiv. 2026 Jun 29:2026.06.24.733515. Preprint. [Version 1] doi: 10.64898/2026.06.24.733515 (PMC13345181; doi:10.64898/2026.06.24.733515)

SUPPLEMENTARY FIGURE LEGENDS

Fig. S1

No significant defect in evoked DA release in the Nucleus Accumbens (NAc) of the ventral striatum of Synj1^{RQ} mice. A. Representative traces of DA release in the NAc evoked by single-pulse stimulation. Traces are normalized to dF max (left). Quantification of the amount of DA released from terminals innervating the NAc in response to single stimulatory events (right). Quantifications are presented as a percentage of dF max (achieved through bath application of 100 µM DA). Synj1^{RQ}: n=6, N=2; *Synj1*$^{+/RQ}$ (control): n=10, N=3.. **B.** Plot illustrating no difference in evoked DA release when release is evoked by consecutive single pulses. Responses are normalized to the first response in the stimulation protocol. *Synj1*$^{+/RQ}$: n=10, N=3. Synj1^{RQ}: n=6, N=2. **C.** Representative traces of DA release in the NAc in response to 5-pulse stimulation at 10 Hz. Traces are normalized to dF max (left). Quantification of the amount of DA released from terminals innervating the NAc in response to a 5-pulse train at 10 Hz. Quantifications are based on the dF of the 5th pulse in the train and are presented as a percentage of dF max (achieved through bath application of 100 µM DA). Synj1^{RQ}: n=6, N=2; *Synj1*$^{+/RQ}$ (control): n=10, N=3. (right). **D.** Deficits in vesicle recycling are absent in the NAc of Synj1^{RQ} mice when release is evoked a 5-pulse train at 10 Hz. Responses are normalized to the first response in the stimulation protocol. *Synj1*$^{+/RQ}$: n=10, N=3. Synj1^{RQ}: n=6, N=2.

Fig. S2

Dystrophic DAergic axon making contacts with multiple cell bodies in the DLS of a delipidated tdTomato-Synj1^{RQ} mouse brain. A. Confocal imaging of a 100 µm thick vibrotome section of delipidated DLS from a Synj1^{RQ} mouse expressing tdTomato in DAergic axons. The large dystrophic DA axon stands out among the fine network of normal DA axons. **B** and **C**. 3 dimensional views of the large dystrophic axon shown in A. **C**. Segmentation of the axon (cyan) shown in A and B, revealing its contacts with 6 cell bodies (dark blue). Scale bars: 20 µm.

Movie S1

3D view of dystrophic DAergic axons distribution in the striatum of a Synj1^{RQ} mouse expressing tdTomato in DAergic neurons. The entire mouse brain had been cleared by delipidation and the tdTomato fluorescence (white) was imaged by light sheet microscopy. Only the striatum, defined by the fluorescence of tdTomato, is shown. Large foci of tdTomato fluorescence were segmented and shown in red superimposed onto the diffuse (white) tdTomato fluorescence corresponding to the normal network of DAergc axons. Note the selective concentration of the large fluorescence foci (dystrophic axon segments) in the dorsal striatum. A small volume of the region most enriched in dystrophic axons is shown at higher magnification in Movie S3. The scattered white spots visible in the ventral striatum represent cell bodies expressing low levels of tdTomato probably as an off-target effect of the DAT promoter used for the study. They stand out in this high contrast movie.

Movie S2

Sequence (rostral to caudal) of coronal views of optical section of the striatum shown in Fig. 1D and in movie S1.

Movie S3

3D view of a small volume of the region most enriched in dystrophic axons from the striatum shown in movie S1 and S2. A 100 µm thick vibratome section was generated from the cleared brain and examined by confocal microscopy. A snapshot of this movie is shown in Fig. 1F. Scale bar: 30 µm.

Movie S4

FIB-SEM stack of a small volume of the striatum of a Synj1^{RQ} mouse obtained with a Zeiss Crossbeam 550 (Yale CCMI). The movie illustrates the two DAergic dystrophic axon segments numbered #2 and #3 in Fig. 4. An astrocyte is pseudocolored in light green. Dystrophic axons are pseudocolored in light magenta and mitochondria in the dystrophc axons in dark green. (Scale bar: 1.5µm)

Movie S5

3D reconstruction of the FIB-SEM volume of the DAergic dystrophic axon segments numbered #2 and #3 in Fig. 4. Green = surface of an astrocyte; blue = blood vessel lumen; pink = surface view of DAergic dystrophic axon segments; magenta = membrane whorls, dark green = mitochondria. Figure 3I-L are snapshots of this movie. (Scale bar: 1 µm)

Movie S6

FIB-SEM stack of a small volume of the striatum of a Synj1^{RQ} mouse at 8 x 8 x 8 nm voxel size with a customized equipment (HHMI Janelia Research Campus). The movie shows that onion-like structures surround the evaginations of a neighboring cell. A red line outlines the outer surface of the dystrophic axon. A cyan line outlines the plasma membrane of the evaginations of the neighboring cell trapped into the dystrophic axon. (Scale bar: 800nm)

Movie S7

3D reconstruction of the structures shown in Movie S6. Only the outer surface of the dystrophic axon terminal (light blue), the membrane whorl (brown) and the invagination of the neigh neighboring cell are shown. (Scale bar: 800nm)

Movie S8

Z stack images of the field shown in Fig. S2A and B. The tdTomato fluorescence is shown in white and the large dystrophic axon that contacts the cell bodies (dark blue) is pseudocolored in cyan.

Movie S9

3D view of the image stack shown in Movie S8.

Movie S10

3D reconstruction of the large dystrophic DAergic axon shown in Fig. S2B and C and in movies S8 and S9. Yellow = saturated fluorescence intensity of tdTomato in the dystrophic DAergic axon; cyan = segmented dystrophic axon; blue = cell bodies contacted by the dystrophic DAergic axon.

Fig.S1

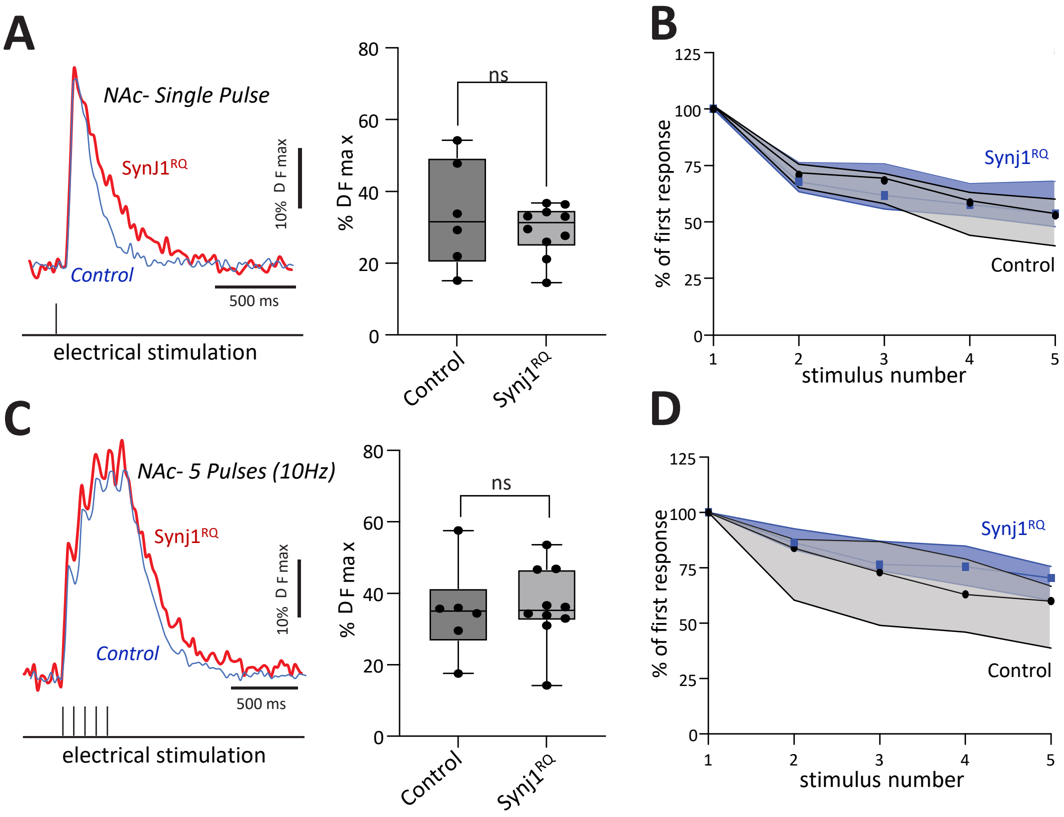
A
NAc- Single Pulse
SynJ1RQ
Control
10% D F max
500 ms
electrical stimulation
% D F ma x
80
60
40
20
0
ns
Control
Synj1RQ
B
% of first response
125
100
75
50
25
0
Synj1RQ
Control
1
2
3
4
5
stimulus number
C
NAc- 5 Pulses (10Hz)
Synj1RQ
Control
10% D F max
500 ms
electrical stimulation
% D F ma x
ns
Control
Synj1RQ
D
% of first response
Synj1RQ
Control
stimulus number

Fig.S2

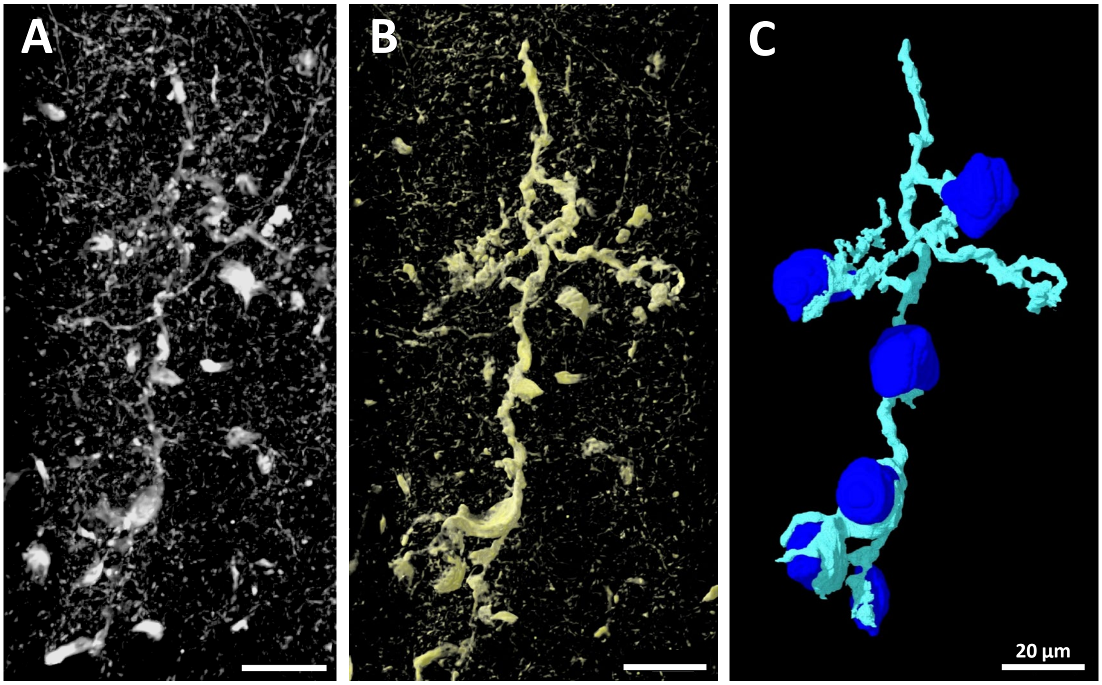

Supplement: Supplement 1 [file media-1.pdf]
